# The cadaver of a Caucasian man with a supernumerary fourth dorsal interosseous muscle in the right hand: a case report

**DOI:** 10.1186/1752-1947-5-393

**Published:** 2011-08-18

**Authors:** Konstantinos Natsis, George Tsakotos, Konstantinos Vlasis, Juergen Koebke

**Affiliations:** 1 Department of Anatomy, Medical School, Aristotle University of Thessaloniki, P.O. Box: 300, Postal Code: 54124, Thessaloniki, Greece; 2 Center of Anatomy, Medical School, University of Cologne, Gebäude 35, Joseph-Stelzmann Str. 9, D-50931 Köln, Germany

## Abstract

**Introduction:**

The human hand is a complex anatomic entity consisting of many muscles, nerves, and vessels, thus providing a special ability to perform accurate and meticulous movements. In this group of muscles are the four dorsal interosseous muscles.

**Case presentation:**

A distinct supernumerary fourth dorsal interosseous muscle was found in the right hand of the cadaver of a 76-year-old Caucasian man without any other concomitant abnormality.

**Conclusions:**

The presence of such an additional muscle in the hand should be considered in the management of hand deformities, whether the treatment is conservative or surgical.

## Introduction

The dorsal interosseous muscles of the hand arise, typically, by two heads from the sides of the adjacent metacarpal bones. Distally, these two heads form a thin tendon and their possible insertion sites are the volar plate and the base of the proximal phalanx, joint capsule, extensor expansion, and transverse and oblique retinacular ligaments and lateral bands of the extensor tendon. The tendon of the first and second dorsal interosseous muscles inserts at the radial aspect of the proximal phalanx of the index and median fingers, respectively, whereas the tendon of the third and fourth interosseous muscles inserts at the ulnar aspect of the proximal phalanx of the median and paramedian fingers, respectively.

In the literature, many anatomic variations of these muscles have been described. Interosseous muscles may consist of one to three heads. According to Eladoumikdachi and colleagues [1], 38% of the palmar interossei and 75% of the dorsal interossei have more than one head. Specifically, the fourth interosseous muscle might have one head in a ratio of 7%, two heads in 71.5%, and three heads in 21.5%, and, as mentioned above, the possible insertion sites are at the proximal phalanx of the paramedian. To the best of our knowledge, the presence of a discrete supernumerary dorsal interosseous muscle that is not a head of the normal fourth interosseous muscle has not been reported [2,3].

## Case presentation

**Figure 1:**
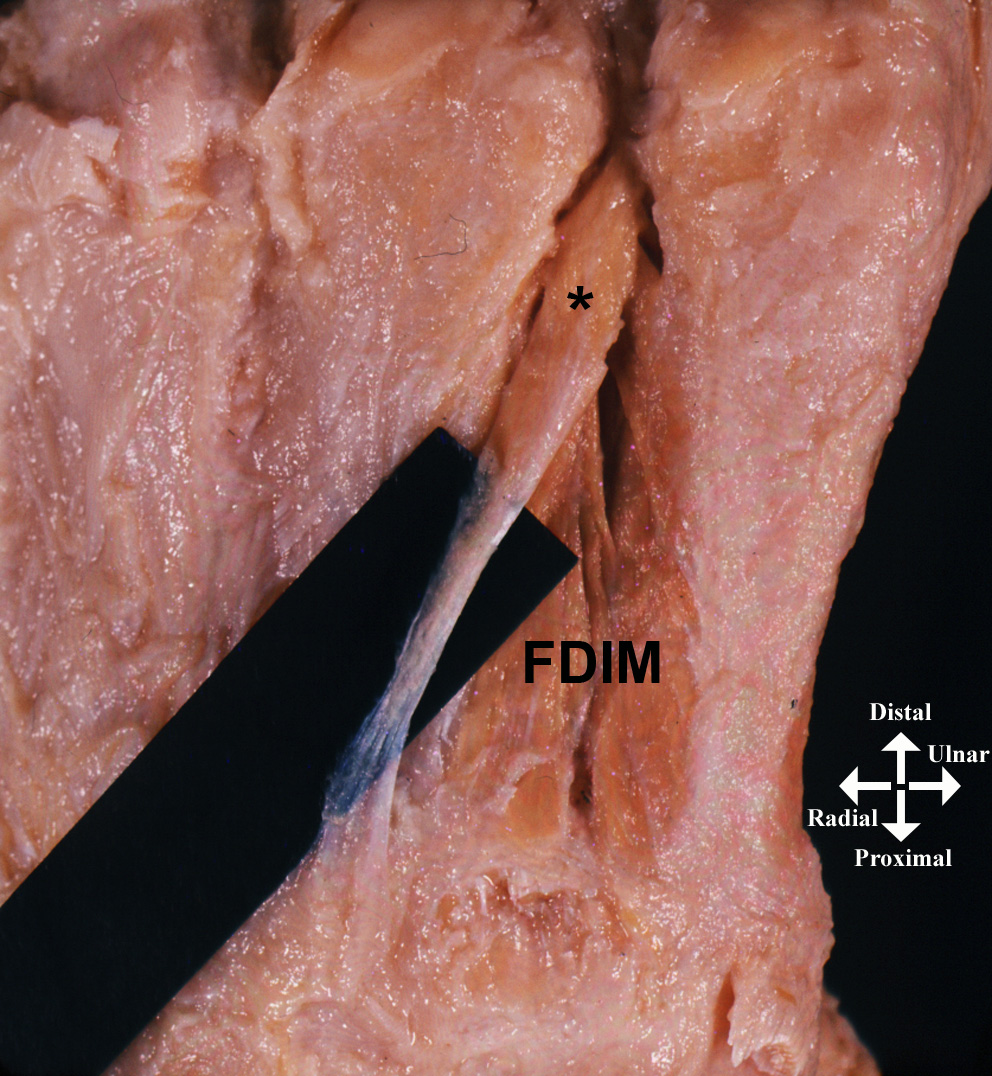
**Dorsal view of the right hand of the cadaver of a 76-year-old man**. *Supernumerary fourth dorsal interosseous muscle. FDIM: (normal) fourth dorsal interosseous muscle.

During a routine cadaveric dissection of the right hand of a 76-year-old Caucasian man, we observed a supernumerary dorsal interosseous muscle. The supernumerary muscle was not a head of the normal fourth dorsal interosseous muscle, which in this case had two heads, but lay superficially to the normal muscle, representing a discrete muscle. The supernumerary muscle arose from the radial surface of the head of the fifth metacarpal, coursed obliquely upward radially and dorsally, and inserted into the dorsal aspect of the fourth metacarpal base (Figure 1). In the other hand, this accessory muscle was absent and no other congenital abnormality was present. The vascular supply was from the dorsal metacarpal artery and innervation from a deep branch of the ulnar nerve. The supernumerary fourth dorsal interosseous muscle was lying deep to the tendons of the extensor digitorum muscle and extensor digiti minimi muscle and did not seem to have affected their course and function.

## Discussion

The presence of a supernumerary fourth dorsal interosseous muscle is usually asymptomatic but may have some clinical implications. Namely, it may alter normal biomechanical behavior during movement of the hand, may affect the functional capacity of intrinsic muscles [4], or may contribute to increased intracompartmental pressures [5]. It might be noticed in laborers who overuse their hands. Also, after a metacarpal fracture, the bony fragments often are dislocated because of traction from the surrounding muscles [6]. One of the most common fractures is of the head of the fifth metacarpal, the so-called "boxer's" fracture, and a hypothetical supernumerary dorsal interosseous muscle may contribute to the effort of its reduction.

## Conclusions

Muscular variations should always be taken into account when a clinician encounters hand deformities, whether the treatment is conservative or surgical. Adequate knowledge of muscular abnormalities is very important for hand surgeons while dealing with fractures, stiff joints, claw toe, or tendon's transfer. Because of its anatomic position and its specific course, the muscle described above can be included in the interosseous muscle variations as a distinct, supernumerary fourth interosseous muscle.

## Consent

Written informed consent was obtained from the patient's relative for publication of this case report and any accompanying images. A copy of the written consent is available for review by the Editor-in-Chief of this journal.

## Competing interests

The authors declare that they have no competing interests.

## Authors' contributions

KN performed the dissection and gave final approval for submitting the manuscript. GT and KV analyzed the data and wrote the manuscript. JK performed the dissection and made the final review. All authors read and approved the final manuscript.
